# A Comparative Study of In-Air Trajectories at Short and Long Distances in Online Handwriting

**DOI:** 10.1007/s12559-017-9501-5

**Published:** 2017-07-27

**Authors:** Carlos Alonso-Martinez, Marcos Faundez-Zanuy, Jiri Mekyska

**Affiliations:** 1 ESUP Tecnocampus (Pompeu Fabra University), Av. Ernest Lluch 32, 08302 Mataró, Spain; 2 Department of Telecommunications, Faculty of Electrical Engineering and Communication, Brno University of Technology, Technicka 10, 616 00 Brno, Czech Republic

**Keywords:** Handwriting, Biometrics, In-air trajectories

## Abstract

Existing literature about online handwriting analysis to support pathology diagnosis has taken advantage of in-air trajectories. A similar situation occurred in biometric security applications where the goal is to identify or verify an individual using his signature or handwriting. These studies do not consider the distance of the pen tip to the writing surface. This is due to the fact that current acquisition devices do not provide height formation. However, it is quite straightforward to differentiate movements at two different heights (a) short distance: height lower or equal to 1 cm above a surface of digitizer, the digitizer provides *x* and *y* coordinates; (b) long distance: height exceeding 1 cm, the only information available is a time stamp that indicates the time that a specific stroke has spent at long distance. Although short distance has been used in several papers, long distances have been ignored and will be investigated in this paper. In this paper, we will analyze a large set of databases (BIOSECUR-ID, EMOTHAW, PaHaW, OXYGEN-THERAPY, and SALT), which contain a total amount of 663 users and 17,951 files. We have specifically studied (a) the percentage of time spent on-surface, in-air at short distance, and in-air at long distance for different user profiles (pathological and healthy users) and different tasks; (b) the potential use of these signals to improve classification rates. Our experimental results reveal that long distance movements represent a very small portion of the total execution time (0.5% in the case of signatures and 10.4% for uppercase words of BIOSECUR-ID, which is the largest database). In addition, significant differences have been found in the comparison of pathological versus control group for letter “l” in PaHaW database (*p* = 0.0157) and crossed pentagons in SALT database (*p* = 0.0122).

## Introduction

Speech and handwriting are probably the most difficult tasks performed by human beings, because they differentiate us from animals. Handwriting requires very fine motor skills, probably more so than speech, because some animals can imitate human sounds but no animal can write. In addition, we learn to speak first and then we learn how to read and write, when the brain is more mature.

Handwriting analysis is a good way to study the human brain in a non-invasive way. This knowledge, once acquired, can be applied to artificial systems that emulate the human brain. We consider that handwriting movements are more complex by far than what has been analyzed in the past. In fact, some parts of the movements have been neglected. With this paper, we will analyze this kind of movements, which will be defined in posterior sections as in-air at long distance. This kind of movements can be used to improve artificial intelligence for biometric applications such as health and security [1–4].

**Fig. 1:**
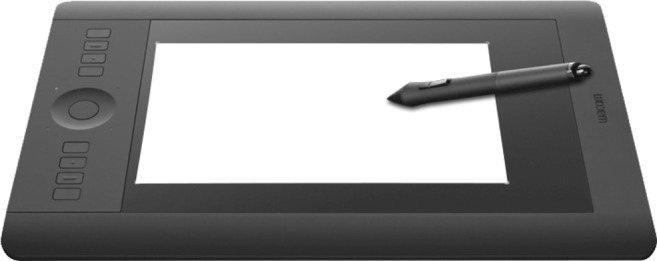
Intuos Pro L digitizing tablet and pen

**Fig. 2:**
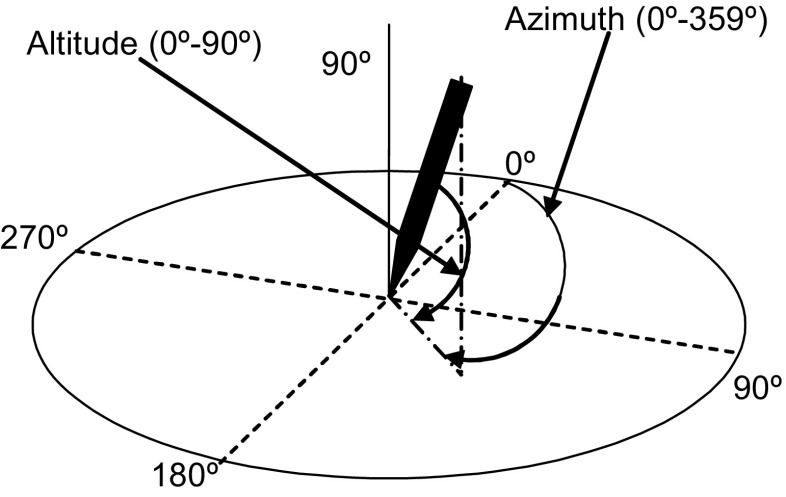
Azimuth and altitude angles of the pen with respect to the plane of the writing surface

In the past, the analysis of handwriting had to be performed in an offline manner. Only the writing itself (strokes on a piece of paper) were available for analysis. Nowadays, modern-capturing devices like digitizing tablets and pens or online whiteboards can gather data without losing its temporal dimension. When spatiotemporal information is available, its analysis is referred to as online. A typical modern-digitizing tablet (Fig. 1) not only gathers the x-y coordinates that describe the movement of the writing device as it changes its position, but it can also collect other data, mainly the pressure exerted by the writing device on the writing surface, the azimuth (the angle of the pen in the horizontal plane), and the altitude (the angle of the pen with respect to the vertical axis) (see (Fig. 2)). From now own, x-y coordinates, pressure, azimuth, and altitude will be referred to as *features of the handwriting*.

A very interesting aspect of the modern online analysis of handwriting is that it can consider information gathered when the writing device was not exerting pressure on the writing surface. Thus, the movements performed by the hand while writing a text can be split into two classes:

1. On-surface trajectories (pen-downs), corresponding to the movements executed while the writing device is touching the writing surface. Each of these trajectories produces a visible stroke. We will call this kind of movement on-surface.
2. In-air trajectories (pen-ups), corresponding to the movements performed by the hand while transitioning from one stroke to the next one. During these movements, the writing device exerts no pressure on the surface. This class can be split into two subsets:
  - In-air at short distances (in-air_S_), when the distance from the tip of the pen to the writing surface is lower or equal to 1 cm. In this case, the digitizing device can track the (*x*, *y*) coordinates during the pen movement.
  - In-air at long distances (in-air_L_), when distances from the tip of the pen to the writing surface are higher than 1 cm. In this case, the digitizing device is not able to track the movements and we only know the time spent at high distance.

In our previous research, we have focused on on-surface and in-air_S_ movements discarding in-air_L_ movements because they do not provide the same amount of data as the previous ones. In fact, the unique parameters are just the number of strokes at long distance and time spent at long distance. For instance, in [5], we applied information theory to demonstrate that on-surface and in-air_S_ contain almost the same amount of information and they are not redundant. This was an important milestone because in-air trajectories had received almost no attention at all, even in online approaches where spatiotemporal information is available.

**Fig. 3:**
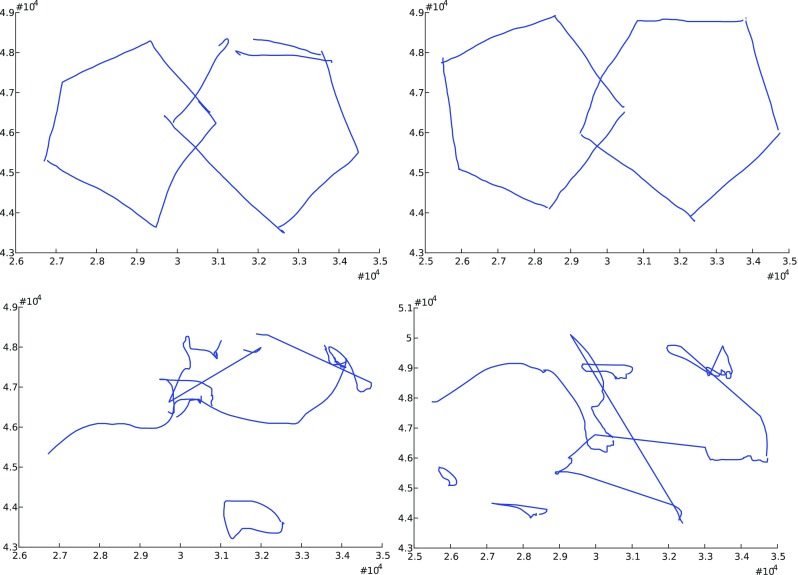
On-surface (top) and in-air (bottom) trajectories from two executions of two crossed pentagons

Figure 3 shows two examples of on-surface and in-air_S_ trajectories taken from two executions of the pentagon test performed by two different writers from the Emothaw database.

**Fig. 4:**
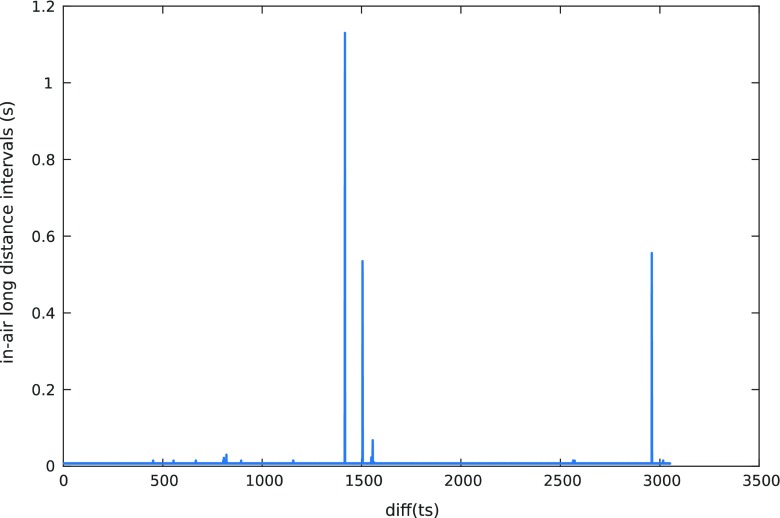
Time stamp difference of consecutive samples for an example of accepted file from PaHaW database task write *lektorka* word twice

**Fig. 5:**
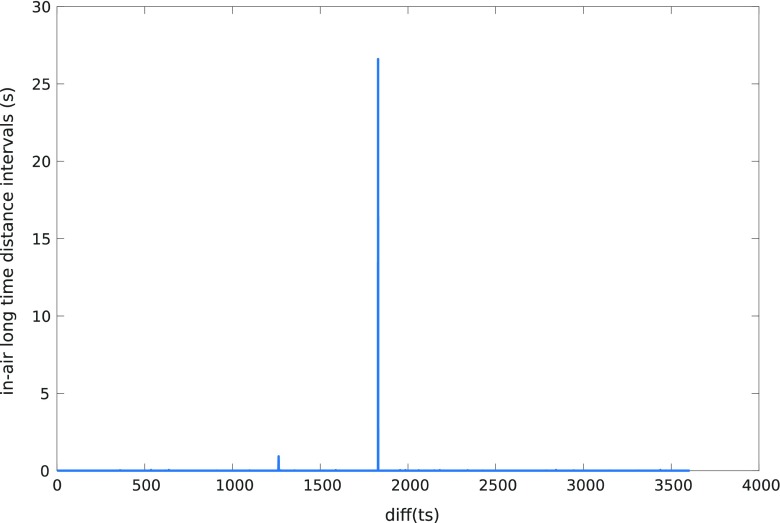
Time stamp difference of consecutive samples for an example of discarded file from PaHaW database task write *lektorka* word twice

In-air_L_ can be detected looking at the time stamp provided by the digitizing tablet. During in-air_L_ time, the tablet is unable to track the tip of the pen and no samples are acquired. Nevertheless, time stamp is increasing and the next time that the pen touches the surface, the samples are stored again in the file and the time jump can be detected. Figure 4 shows the difference of consecutive time stamps for an example file. For most of the samples (on-surface and in-air_S_), this value is small (typically two units). However, there are some peaks, which correspond to in-air_L_ movements. Figure 4 reveals 11 strokes of the type in-air_L_. Sometimes, this time is abnormally long. This is probably due to some acquisition problem, where the user started to speak with the database acquisition supervisor for minutes. We will label these cases and will not include them in the average computation of time spent at in-air_L_. We consider these cases when time in-air_L_ is greater than 70% of the total time. In particular, we have found this phenomenon in 5 files from the analyzed databases (total amount of analyzed files is 17,951 files) (e.g. see Fig. 5).

## Experimental Databases

In this paper, we have analyzed a set of different databases that contain different tasks and user profiles. The databases share the existence of handwritten tasks. In this section, we will summarize the main characteristics of the analyzed databases.

### BIOSECUR-ID

This database is a multimodal biometric one and includes eight biometric traits: speech, iris, face (still images and videos), handwritten signature and handwritten text, fingerprints, hand, and keystroking. This database acquired inside the Biosecur-ID project was developed by a consortium of six Spanish Universities, more details can be found in [6]. With respect to handwriting and signatures, this database defines five different tasks: a Spanish text in lower-case, ten digits written separately, 16 Spanish words in upper-case, four genuine signatures, and one forgery of the three precedent subjects.

### EMOTHAW

As described in [7], this database includes samples of 129 participants who are classified on the basis of their emotional states: anxiety, depression, and stress or health. This classification is assessed by the Depression–Anxiety–Stress Scales (DASS) questionnaire. Seven tasks are recorded through a digitizing tablet: pentagons and house drawing, words in capital letters copied in handprint, circles with left and right hand, clock drawing, and one sentence copied in cursive writing.

### PAHAW

The Parkinson’s Disease Handwriting Database (PaHaW) consists of multiple handwriting samples from 37 Parkinson’s disease patients, and 38 gender and age matched controls. Eight different tasks were recorded through a digitizing tablet: spiral drawing, letters, words, and a sentence. The details about this database can be found in [8].

### OXIGEN-THERAPY

This database described in [9] includes eight patients with hypoxemia who performed two tasks: house and clock drawing, before and after breathing 30 min with O_2_ with the aim of evaluating changes in psychomotor functions.

### SALT

As described in [10], the database includes samples of 52 participants: 23 with Alzheimer’s disease, 12 with mild cognitive impairment (MCI), and 17 healthy controls. Seven tasks were recorded: crossed pentagons, spiral, 3D house, clock drawings, spontaneous, copied, and dictated handwriting.

## Experimental Results

The first experiments consisted of analyzing the three kinds of time in absolute and relative values as well as the number of strokes in all the scenarios. Tables 1, 2, 3, 4, and 5 summarize the results for the analyzed databases. It is worth remarking that different databases contain different tasks described in the previous section.

**Table 1:** BIOSECUR-ID database. Time in absolute units and relative time in parenthesis

	Time			Strokes		
Task	On-surface	In-air_S_	In-air_L_	On-surface	In-air_S_	In-air_L_
Genuine signature	2857.6 (79.6%)	715.4 (19.9%)	17.5 (0.5%)	6.62	5.94	0.32
Skilled forgeries	5447.9 (68.5%)	2373.4 (29.9%)	128.5 (1.6%)	6.58	6.21	0.63
Lower case words	110,445.1 (55.9%)	76,454 (38.7%)	10,644.4 (5.4%)	335.01	367.16	33.16
Numbers	3677.3 (53.6%)	3071.1 (44.7%)	117.0 (1.7%)	11.66	11.46	0.79
Uppercase words	73,608.8 (54.4%)	47,756.2 (35.3%)	14,073.4 (10.4%)	313.49	343.29	30.81

**Table 2:** EMOTHAW database. Time in absolute units and relative time in parenthesis

	Time			Strokes		
Task	On-surface	In-air_S_	In-air_L_	On-surface	In-air_S_	In-air_L_
a. Depression						
Two-pentagon	11394.0 (55.5%)	7755.8 (37.7%)	1393.3 (6.8%)	9.26	13.15	9.47
House	18765.4 (53.6%)	13933.1 (39.8%)	2329.4 (6.7%)	23.74	33.00	20.97
Capital letters	15789.4 (51.0%)	13112.3 (42.4%)	2050.1 (6.6%)	59.79	65.91	12.15
Loops with left hand	10183.9 (97.7%)	215.8 (2.1%)	21.3 (0.2%)	1.26	0.41	0.21
Loops with right hand	8542.7 (98.9%)	58.6 (0.7%)	39.3 (0.4%)	1.18	0.21	0.06
Clock	14228.8 (45.0%)	14905.2 (47.2%)	2468.7 (7.8%)	27.35	36.91	21.44
Sentence	15288.8 (60.4%)	8052.4 (31.8%)	1958.5 (7.8%)	41.24	47.41	11.09
b. Stress						
Two-pentagon	11283.0 (55.0%)	7768.6 (37.9%)	1444.1 (7.1%)	9.41	13.89	11.39
House	18868.4 (52.5%)	14378.6 (40.0%)	2685.6 (7.5%)	25.45	35.14	21.32
Capital letters	15732.3 (50.1%)	13555.7 (43.1%)	2135.2 (6.8%)	60.80	67.09	12.04
Loops with left hand	10648.5 (97.3%)	233.5 (2.1%)	66.6 (0.6%)	1.59	0.77	0.95
Loops with right hand	9264.1 (99.3%)	40.0 (0.4%)	23.9 (0.3%)	1.13	0.14	0.04
Clock	14481.5 (44.8%)	14934.1 (46.2%)	2896.2 (9.0%)	27.63	37.80	21.41
Sentence	15756.6 (59.4%)	8539.8 (32.2%)	2215.8 (8.4%)	42.55	48.95	10.84
c. Anxiety						
Two-pentagon	11474.7 (57.3%)	7135.2 (35.6%)	1420.7 (7.1%)	8.70	12.75	10.16
House	18871.9 (53.6%)	13683.5 (38.8%)	2672.7 (7.6%)	23.77	32.89	18.95
Capital letters	16010.0 (50.9%)	13356.9 (42.5%)	2082.7 (6.6%)	60.48	66.39	10.96
Loops with left hand	10248.4 (96.9%)	224.3 (2.1%)	103.0 (1.0%)	1.57	0.79	0.96
Loops with right hand	8793.2 (99.3%)	35.6 (0.4%)	23.9 (0.3%)	1.11	0.13	0.04
Clock	14175.3 (46.5%)	13487.9 (44.3%)	2811.3 (9.2%)	26.27	35.48	19.54
Sentence	15676.5 (59.9%)	8402.2 (32.1%)	2107.5 (8.0%)	41.96	48.14	10.38
d. Control						
Two-pentagon	10256.0 (49.7%)	8670.5 (42.1%)	1684.9 (8.2%)	10.13	15.27	12.91
House	17468.1 (49.0%)	15150.2 (42.5%)	3044.5 (8.5%)	26.63	36.23	22.27
Capital letters	15699.2 (48.9%)	13721.2 (42.8%)	2677.1 (8.3%)	61.46	67.84	11.68
Loops with left hand	9737.1 (98.5%)	133.3 (1.3%)	17.7 (0.2%)	1.18	0.30	0.41
Loops with right hand	8992.1 (98.4%)	123.2 (1.3%)	23.9 (0.3%)	1.07	0.09	0.04
Clock	12365.6 (38.9%)	16180.8 (50.9%)	3229.5 (10.2%)	27.13	37.25	22.63
Sentence	15660.0 (53.6%)	9539.6 (32.6%)	4024.3 (13.8%)	42.41	49.43	11.43

**Table 3:** PAHAW database. Time in absolute units and relative time in parenthesis

	Time			Strokes		
Task	On-surface	In-air_S_	In-air_L_	On-surface	In-air_S_	In-air_L_
a. Control						
Spiral	18,665.8 (98.6%)	171.5 (0.9%)	103.2 (0.5%)	1.40	1.97	1.94
Letter *l*	8077.8 (57.6%)	3868.3 (27.6%)	2069.6 (14.8%)	5.21	18.16	15.50
Bigram *le*	10,545.9 (71.2%)	2998.4 (20.2%)	1274.3 (8.6%)	5.13	14.03	11.00
Word *les*	12,309.1 (69.2%)	3513.0 (19.7%)	1977.7 (11.1%)	5.29	15.11	11.82
Word *lektorka*	14,931.2 (73.0%)	3238.1 (15.9%)	2279.8 (11.1%)	7.00	16.97	12.00
Word *porovnat*	13,071.5 (74.4%)	3356.7 (19.1%)	1139.4 (6.5%)	8.08	18.08	11.82
Word *nepopadnout*	8757.5 (83.8%)	1512.5 (14.5%)	179.0 (1.7%)	5.29	8.47	4.50
Sentence	14,481.3 (58.4%)	7457.9 (30.1%)	2844.6 (11.5%)	15.24	31.87	19.34
b. Parkinson patients						
Spiral	24,057.4 (95.4%)	618.3 (2.4%)	536.6 (2.2%)	2.03	6.78	7.31
Letter *l*	8928.1 (63.8%)	4132.5 (29.5%)	939.1 (6.7%)	5.51	16.08	12.59
Bigram* le*	12,143.2 (69.1%)	4094.1 (23.3%)	1330.4 (7.6%)	5.57	17.08	13.76
Word* les*	14,702.7 (69.6%)	4093.1 (19.4%)	2330.9 (11.0%)	5.76	19.22	15.54
Word *lektorka*	17,716.2 (76.3%)	36,045.0 (15.5%)	1890.1 (8.2%)	7.22	17.97	12.49
Word *porovnat*	14,690.6 (75.8%)	3808.9 (19.6%)	891.1 (4.6%)	8.86	18.11	11.00
Word *nepopadnout*	9784.0 (79.8%)	2115.7 (17.2%)	365.6 (3.0%)	6.76	11.30	5.86
Sentence	16,176.5 (58.2%)	8252.3 (29.9%)	3300.1 (11.9%)	16.57	36.81	23.62

**Table 4:** OXIGEN-THERAPY database. Time in absolute units and relative time in parenthesis

	Time			Strokes		
Task	On-surface	In-air_S_	In-air_L_	On-surface	In-air_S_	In-air_L_
a. Before O_2_						
House	32,699.0 (49.6%)	22,184.8 (33.7%)	11,033.8 (16.7%)	28.88	131.13	141.29
Clock	20,144.0 (40.2%)	21,824.0 (43.6%)	8104.3 (16.2%)	27.25	94.13	79.00
b. After O2						
House	26,572.1 (53.6%)	18,429.1 (37.1%)	4606.5 (9.3%)	27.70	74.57	51.96
Clock	16,619.8 (46.4%)	16,007.8 (44.7%)	3197.6 (8.9%)	25.21	57.21	37.29

**Table 5:** SALT database. Time in absolute units and relative time in parenthesis

	Time			Strokes		
Task	On-surface	In-air_S_	In-air_L_	On-surface	In-air_S_	In-air_L_
a. DCLI						
Crossed pentagons	18,292.8 (60.2%)	8497.3 (27.9%)	3612.6 (11.9%)	10.00	20.33	27.50
Spiral	8219.3 (99.0%)	26.75 (0.3%)	60.8 (0.7%)	1.42	1.75	2.25
3D house	33,503.83 (52.0%)	19,388.6 (30.1%)	11,534.3 (17.9%)	29.50	49.17	50.42
Clock	18,931.9 (31.2%)	24,807.2 (40.9%)	16,917.0 (27.9%)	26.67	52.17	70.50
Spontaneous sentence	16,500.3 (48.8%)	14,322.9 (42.4%)	2966.5 (8.8%)	40.67	47.75	15.33
Sentence copied	26,535.4 (49.3%)	21,918.3 (40.7%)	5404.9 (10.0%)	57.58	69.08	29.58
Sentence dictation	20,710.7 (59.1%)	11,717.8 (33.4%)	2633.0 (7.5%)	43.25	50.08	16.33
b. Alzheimer						
Crossed pentagons	21,535.4 (48.4%)	15,430.1 (34.6%)	7555.4 (17.0%)	14.05	28.00	48.14
Spiral	11,312.2 (88.7%)	1108.8 (8.7%)	327.2 (2.6%)	1.71	1.67	2.52
3D house	40,341.6 (47.3%)	30,465.8 (35.8%)	14,386.2 (16.9%)	31.55	55.23	75.77
Clock	24,524.7 (36.1%)	33,060.4 (48.6%)	10,420.8 (15.3%)	29.36	48.41	50.95
Spontaneous sentence	19,555.9 (48.6%)	17,090.1 (42.4%)	3606.1 (9.0%)	37.23	44.05	17.09
Sentence copied	34,023.8 (45.1%)	33,451.3 (44.4%)	7951.2 (10.5%)	54.32	69.50	35.95
Sentence dictation	26,640.6 (52.7%)	20,723.6 (41.0%)	3189.6 (6.3%)	44.27	54.86	20.45
c. Control						
Crossed pentagons	17,077.7 (50.1%)	13,085.8 (38.4%)	3918.6 (11.5%)	11.88	36.47	36.65
Spiral	6198.3 (91.0%)	426.6 (5.4%)	251.3 (3.6%)	1.63	3.06	2.94
3D house	29,170.5 (43.3%)	26,094.5 (38.7%)	12,152.4 (18.0%)	30.82	72.24	68.12
Clock	18,986.1 (30.2%)	31,299.1 (49.8%)	12,547.1 (20.0%)	29.94	71.38	71.06
Spontaneous sentence	14,990.5 (43.8%)	14,648.8 (42.8%)	4566.4 (13.4%)	35.41	56.88	31.12
Sentence copied	24,684.2 (45.5%)	23,968.8 (44.1%)	5654.8 (10.4%)	53.59	78.00	37.53
Sentence dictation	19,531.1 (56.9%)	13,131.1 (38.2%)	1676.5 (4.9%)	38.71	50.24	16.76

For a given user, the number of strokes is an integer number. However, the table shows the average number of strokes for a specific database and task (in addition to the number of strokes done by the whole set of users split by the number of users). This number is not integer anymore.

Experimental results of BIOSECUR-ID database, which is the largest one according to the number of users and files, reveal that in-air_L_ is almost negligible in the case of signatures, but interestingly, it is three times larger for skilled forgeries than for genuine signatures. For uppercase words, the time in-air_L_ is larger than for the other tasks but still quite modest (10.4%). Thus, this kind of movement is less important than the other two and can probably be ignored without sacrificing a lot of information. For the other databases, a statistical test will be performed after presenting the experimental results.

From all the databases related to diseases, we computed the Mann-Whitney *U* test between study and control groups to determine the existence of statistically significant difference (*p* < 0.05) in the studied features (time and strokes). The results are shown in Table 6.

**Table 6:** EMOTHA (Mann-Whitney *U* test)

Task	*p* T_S_	*p* T_AS_	*p* T_AL_	*p* strokes_S_	*p* strokes_AS_	*p* strokes_AL_
a. Depression/control						
Two-pentagon	0.4316	0.3082	0.0589	0.1374	0.0731	0.0561
House	0.7329	0.0495	0.5002	0.0315	0.0774	0.4217
Capital letters	0.5771	0.5771	0.8744	0.0904	0.2317	0.5994
Loops with left hand	0.7613	0.2380	0.1292	0.2954	0.2742	0.1542
Loops with right hand	0.6592	0.5316	0.7322	0.5067	0.5005	0.7322
Clock	0.1267	0.6293	0.2196	0.6641	0.7739	0.9688
Sentence	0.8992	0.3273	0.1849	0.3794	0.2870	0.5764
b. Anxiety/control						
Two-pentagon	0.2429	0.1020	0.1678	0.1546	0.1010	0.1505
House	0.4564	0.0417	0.4086	0.0652	0.1777	0.2550
Capital letters	0.3770	0.6374	0.3503	0.1731	0.1888	0.7751
Loops with left hand	0.7711	0.1575	0.1723	0.1560	0.1429	0.2017
Loops with right hand	0.9374	1	1	0.9822	0.9762	1
Clock	0.0414	0.1540	0.2410	0.4462	0.5801	0.8294
Sentence	0.7234	0.4259	0.1296	0.5392	0.3971	0.2913
c. Stress/control						
Two-pentagon	0.5665	0.4886	0.3429	0.6173	0.4131	0.3678
House	0.5221	0.2565	0.4705	0.5562	0.7621	0.9188
Capital letters	0.4741	0.9907	0.7934	0.2769	0.4367	0.4662
Loops with left hand	0.3859	0.1625	0.2801	0.1466	0.1498	0.3173
Loops with right hand	0.4795	0.7184	1	0.6875	0.6875	1
Clock	0.0241	0.7401	0.4670	0.6199	0.6496	0.6623
Sentence	0.6819	0.5753	0.1011	0.7034	0.5335	0.4764

*T*_*S*_ time on-surface, *T*_*AS*_ time in-air_S_, *T*_*AL*_ time in-air_L_, *Strokes*_*S*_ strokes on-surface, *Strokes*_*AS*_ strokes in-air_s_, *Strokes*_*AL*_ strokes in-air_L_

We can observe in Table 6 (a. Depression/control) how in crossed pentagon task, the values are very close to the threshold for long distance time and strokes. In house draw, the near time and on-surface strokes show statistical significance. In Table 6 (b. Anxiety/control), house draw shows again that near-distance time is statistically signficant. Finally, in Table 6 (c. Stress/control), we obtain *p* < 0.05 for on-surface time in clock draw only.

As is shown in Table 7, for PaHaW database we obtain statistically significant results in letter *l* long distance time and in bigram *le* for near-distance time and on-surface strokes.

**Table 7:** PaHaW (Mann-Whitney *U* test)

Task	*p* T_S_	*p* T_AS_	*p* T_AL_	*p* strokes_S_	*p* strokes_AS_	*p* strokes_AL_
a. Parkinson/control						
Spiral	0.3947	0.5621	0.0939	0.2857	0.0919	0.0949
Letter *l*	0.4614	0.5529	0.0157	0.2390	0.3611	0.2718
Bigram *le*	0.3015	0.0403	0.5671	0.0090	0.1173	0.1710
Word *les*	0.3015	0.3166	0.6601	0.2941	0.2453	0.4385
Word *lektorka*	0.5166	0.9440	0.3019	0.8111	0.6928	0.4744
Word *porovnat*	0.3878	0.7226	0.4025	0.3778	0.9239	0.7963
Word *nepopadnout*	0.5780	0.1776	0.2836	0.0630	0.1287	0.2538
Sentence	0.2000	0.5850	0.9612	0.3229	0.2720	0.5773

*T*_*S*_ time on-surface, *T*_*AS*_ time in-air_S_, *T*_*AL*_ time in-air_L_, *Strokes*_*S*_ strokes on-surface, *Strokes*_*AS*_ strokes in-air_s_, *Strokes*_*AL*_ strokes in-air_L_

In OXYGEN THERAPY database, the times and number of strokes do not show statistical significance and do not seem to offer a valid classification pattern between pre- and post-O_2_ results (Table 8).

**Table 8:** OXYGEN THERAPY (Mann-Whitney *U* test)

Task	*p* T_S_	*p* T_AS_	*p* T_AL_	*p* strokes_S_	*p* strokes_AS_	*p* strokes_AL_
a. Pre/post O_2_						
House	0.8968	0.8764	0.9174	0.9174	0.8968	0.8968
Clock	0.9218	0.8936	0.9077	0.8665	0.8795	0.8795

*T*_*S*_ time on-surface, *T*_*AS*_ time in-air_S_, *T*_*AL*_ time in-air_L_, *Strokes*_*S*_ strokes on-surface, *Strokes*_*AS*_ strokes in-air_s_, *Strokes*_*AL*_ strokes in-air_L_

In Table 9 (SALT, a. Alzheimer/control), we can observe how on crossed pentagons draw, statistical significance can be found in on-surface time and long distance time. Also, on-surface time presents significance on the sentence copied. No results with *p* < 0.05 were obtained for mild cognitive impairment (MCI)/control (Table 9, b).

**Table 9:** SALT (Mann-Whitney *U* test)

Task	*p* T_S_	*p* T_AS_	*p* T_AL_	*p* strokes_S_	*p* strokes_AS_	*p* strokes_AL_
a. Alzheimer/control						
Crossed pentagons	0.0303	0.1609	0.0122	0.3941	0.6604	0.0891
Spiral	0.0063	0.5132	0.1995	0.9185	0.1338	0.1869
3D house	0.0677	0.1370	0.1297	0.3493	0.7533	0.0720
Clock	0.1071	0.1984	0.1785	0.5526	0.2033	0.6256
Spontaneous sentence	0.1878	0.0524	0.9210	0.3875	0.8316	0.8761
Sentence copied	0.0096	0.1080	0.1096	0.5612	0.3954	0.2629
Sentence dictation	0.0132	0.0721	0.0920	0.2510	0.3953	0.1604
b. MCI/control						
Crossed pentagons	0.1915	0.4925	0.1915	0.2758	0.1688	0.5643
Spiral	0.0968	0.5358	0.0865	0.4290	0.0889	0.0973
3D house	0.2069	0.5500	0.3879	0.6729	0.7734	0.5206
Clock	0.4437	0.9445	0.0738	1	0.8892	0.2854
Spontaneous sentence	0.5500	0.8421	0.9119	0.4124	0.5496	0.6094
Sentence copied	0.1501	0.4384	0.7735	0.2777	0.6260	0.8075
Sentence dictation	0.3640	0.7398	0.2878	0.3407	0.4784	0.5203

*T*_*S*_ time on-surface, *T*_*AS*_ time in-air_S_, *T*_*AL*_ time in-air_L_, *Strokes*_*S*_ strokes on-surface, *Strokes*_*AS*_ strokes in-air_s_, *Strokes*_*AL*_ strokes in-air_L_

## Discussion

Although most of the results in previous tables are not significant, even for on-surface and in-air_S_ information, we should point out that this kind of measurements offers a large set of features that can be extracted, such as speed and acceleration of trajectories and complexity measurements extracted from coordinates *x*, *y*. In fact, a classifier would not be based on a single measurement. It will take advantage of a set of measurements. Thus, high *p* values for on-surface and in-air_S_ do not imply the impossibility to perform a classification. These values are provided just for comparison purpose with in-air_L_ values. In-air_L_ extracted features are limited to time and number of strokes. Thus, the analysis of relevance of this information is simpler.

Nevertheless, this paper points out the tasks and pathologies where more potential improvements can be achieved, because in some tasks, *p* < 0.05 has been obtained.

Looking at the experimental results of pathologies, we can establish that in-air_L_ movements are not relevant but there are some exceptions: crossed pentagon task for depression patients in EMOTHAW, which is near significance (*p* = 0.0589 for time and *p* = 0.0561 for strokes), letter *l* task for PaHaW database (*p* = 0.0157 for time), and crossed pentagons task for Alzheimer/control comparison (*p* = 0.0122 for time). We consider these results especially interesting because crossed pentagons are a very useful measurement in pathological analysis, in fact, it is the only drawing that subjects must perform in the well-established mini-mental state examination, also known as the Folstein test [11].

## Conclusions

One of the main goals of this paper was to study if in-air_L_ information can be discarded in handwritten tasks analysis. Looking at the experimental results, we can conclude that little time is spent by healthy writers at long distance so most of the information is contained on-surface and in-air_S_ distances. This implies that the development of a new acquisition device able to track *x* and *y* coordinates and long distances will probably not be very useful, because few samples will be acquired in this condition. However, experimental results reveal that time spent at long distance is more than three times higher for skilled forgeries than for genuine signatures. This opens a possible research line in security biometrics. A similar consideration can be established for the number of strokes, which is doubled in the case of skilled forgeries with respect to short distance in-air movements. Thus, the existence of long distance movements can be indicative of a signature forgery.

On the other hand, when looking at pathologies, we have found statistically significant differences in the pentagon tasks for Alzheimer/control comparison. This result opens the possibility of investigating in-air at long distance movements further.
