# Correction to: A Comparative Study of In-Air Trajectories at Short and Long Distances in Online Handwriting

**DOI:** 10.1007/s12559-018-9572-y

**Published:** 2018-06-22

**Authors:** Carlos Alonso-Martinez, Marcos Faundez-Zanuy, Jiri Mekyska

**Affiliations:** 1 ESUP Tecnocampus (Pompeu Fabra University), Av. Ernest Lluch 32, 08302 Mataró, Spain; 2 Department of Telecommunications, Faculty of Electrical Engineering and Communication, Brno University of Technology, Technicka 10, 616 00 Brno, Czech Republic

**Correction to: Cogn Comput (2017) 9(5):712–720**

10.1007/s12559-017-9501-5

The article A Comparative Study of In-Air Trajectories at Short and Long Distances in Online Handwriting, written by Carlos Alonso-Martinez, Marcos Faundez-Zanuy and Jiri Mekyska, was originally published electronically on the publisher’s internet portal (currently SpringerLink) on 27 July 2017 without open access. With the author(s)’ decision to opt for Open Choice the copyright of the article changed on June 2018 to © The Author(s) 2018 and the article is forthwith distributed under the terms of the Creative Commons Attribution 4.0 International License (http://creativecommons.org/licenses/by/4.0/), which permits use, duplication, adaptation, distribution and reproduction in any medium or format, as long as you give appropriate credit to the original author(s) and the source, provide a link to the Creative Commons license and indicate if changes were made.

The original article has been corrected.

